# Ozone‐induced eosinophil recruitment to airways is altered by antigen sensitization and tumor necrosis factor‐*α* blockade

**DOI:** 10.14814/phy2.13538

**Published:** 2017-12-15

**Authors:** Sarah A. Wicher, Katy L. Lawson, David B. Jacoby, Allison D. Fryer, Matthew G. Drake

**Affiliations:** ^1^ Department of Physiology and Pharmacology Oregon Health & Sciences University Portland Oregon; ^2^ Division of Pulmonary and Critical Care Medicine Department of Medicine Oregon Health & Science University Portland Oregon

**Keywords:** Airway, asthma, eosinophil, nerve, ozone, TNF*α*

## Abstract

Ozone is an atmospheric pollutant that causes lung inflammation and airway hyperresponsiveness. Ozone's effects occur in two distinct phases that are mediated by different populations of eosinophils. In the acute phase 1 day after exposure, mature airway‐resident eosinophils alter parasympathetic nerve function that results in airway hyperresponsiveness. At this time point, the severity of hyperresponsiveness correlates with the number of eosinophils in close proximity to airway nerves, but not with eosinophils in bronchoalveolar lavage. Three days later, newly divided eosinophils are recruited to airways by a tumor necrosis factor‐*α*‐dependent mechanism. These new eosinophils paradoxically attenuate ozone‐induced airway hyperresponsiveness. Ozone's effects on airway tissue eosinophils and nerve‐associated eosinophils 3 days after exposure are unknown. Thus, we tested ozone's effects on eosinophils in airway subepithelium and around airway nerves 1 and 3 days after ozone in nonsensitized and ovalbumin‐sensitized guinea pigs with or without the tumor necrosis factor‐*α* antagonist, etanercept, and compared changes in eosinophils with ozone‐induced airway hyperresponsiveness. More eosinophils were present in small, noncartilaginous airways and along small airway nerves compared to large cartilaginous airways in all treatment groups. The number of airway and nerve‐associated eosinophils were unaffected 1 day after ozone exposure, whereas significantly fewer airway eosinophils were present 3 days later. Airway and nerve‐associated eosinophils were also decreased in small airways 3 days after ozone in sensitized animals. These changes were blocked by etanercept. Airway eosinophils, but not nerve‐associated or bronchoalveolar lavage eosinophils correlated with airway hyperresponsiveness 3 days after ozone. Our findings indicate ozone causes persistent alterations in airway eosinophils and reinforce the importance of characterizing eosinophils’ effects within distinct airway compartments.

## Introduction

Ground‐level ozone is an environment pollutant with a substantial global burden. Millions of people are exposed to unsafe levels, leading to an estimated 700,000 premature deaths annually worldwide (Anenberg et al. 2010). When inhaled, ozone causes airway hyperresponsiveness in both healthy humans (Golden et al. 1978; Holtzman et al. 1979; Aris et al. 1993) and in asthmatics (Peters et al. 2001; Ierodiakonou et al. 2016), as well as in animals (Gordon et al. 1984; Roum and Murlas 1984; Boushey and Holtzman 1985; Stevens et al. 1995; Moore et al. 2012; Verhein et al. 2013; Yang et al. 2016). Asthmatics are at especially high risk for ozone‐induced respiratory complications due to their heightened inflammatory and bronchoconstrictive responses (Peden et al. 1997). Consequently, asthma‐related emergency department visits and hospitalizations increase significantly 1–3 days after high ozone days (Mar and Koenig 2009; Gleason et al. 2014; Sheffield et al. 2015; Lam et al. 2016).

Ozone‐induced airway hyperresponsiveness has an acute and lag phase (Mar and Koenig 2009; Gleason et al. 2014; Sheffield et al. 2015), which, in guinea pigs, is associated with changes in eosinophil populations in the lungs (Schultheis and Bassett 1994; Yost et al. 1999, 2005; Wicher et al. 2017). In the acute phase, 1 day after exposure, airway hyperresponsiveness is caused by eosinophils resident in airways. These eosinophils are mature cells that have not recently divided since they do not stain for the thymidine analog (5‐bromo‐2′‐deoxyuridine; BrdU) that incorporates into DNA of all dividing cells after ozone exposure. Mature, airway resident eosinophils cause airway hyperresponsiveness by altering parasympathetic nerve function (Holtzman et al. 1979; Beckett et al. 1985; Schultheis et al. 1994; Yost et al. 1999; Wicher et al. 2017). Under normal conditions, parasympathetic nerves release acetylcholine that activates M_3_ muscarinic receptors on airway smooth muscle to induce bronchoconstriction (Nadel and Barnes 1984; Roffel et al. 1990; Eglen 1996; Kesler and Canning 1999). Simultaneously, acetylcholine activates presynaptic parasympathetic inhibitory M_2_ muscarinic receptors that limit additional acetylcholine release (Fryer and Maclagan 1984). Ozone exposure provokes release of eosinophil major basic protein, which is an endogenous M_2_ receptor antagonist (Jacoby et al. 1993), and M_2_ receptors’ auto‐inhibitory feedback is lost (Gambone et al. 1994; Yost et al. 1999, 2005). Loss of neuronal M_2_ receptor function results in more acetylcholine release and increased bronchoconstriction (Jacoby et al. 1993; Schultheis et al. 1994; Yost et al. 1999, 2005). Airway hyperresponsiveness can be prevented 1 day after exposure by depleting eosinophils with an antibody to interleukin‐5 (AbIL5), or reversed by neutralizing major basic protein with antibody or with heparin. These treatments protect or restore, respectively, neuronal M_2_ receptor function (Gambone et al. 1994; Yost et al. 1999, 2005). Thus, ozone‐induced airway hyperresponsiveness is mediated acutely by eosinophil major basic protein and blockade of inhibitory M_2_ receptors on parasympathetic nerves.

In contrast, eosinophil depletion with AbIL5 3 days after ozone not only fails to prevent hyperresponsiveness, but significantly worsens it (Yost et al. 2005; Wicher et al. 2017). Eosinophils exert a protective effect by day 3 due to an influx of newly divided, BrdU‐positive eosinophils that attenuate airway hyperresponsiveness (Wicher et al. 2017). Tumor necrosis factor‐alpha (TNF*α*) is the critical cytokine that mediates expansion of newly divided protective eosinophils in bone marrow after ozone (Wicher et al. 2017). Consequently, blocking TNF*α* with etanercept prevents the influx of new eosinophils into lungs and significantly worsens ozone‐induced airway hyperresponsiveness 3 days after exposure (Wicher et al. 2017).

Inflammatory responses to ozone differ in atopic asthmatics compared to nonatopics, and in antigen‐sensitized animals. In nonatopic subjects, ozone increases neutrophils in bronchoalveolar lavage (Vagaggini et al. 2010; Kim et al. 2011), whereas significant eosinophilia occurs in atopic asthmatics (Vagaggini et al. 2002; Khatri et al. 2009; Dokic and Trajkovska‐Dokic 2013). In guinea pigs, antigen sensitization prevents development of new, protective eosinophils in bone marrow after ozone, and their beneficial effects on airway function are lost (Wicher et al. 2017). Blocking TNF*α* also has no effect on ozone‐mediated airway hyperresponsiveness in sensitized animals. These differences are relevant given that nearly half of adult asthmatics are sensitized to allergens (Pearce et al. 1999; Salo et al. 2014).

More eosinophils are associated with airway nerves than in any other lung compartment in asthma (Costello et al. 1997). In animal models of airway hyperresponsiveness, the number of eosinophils surrounding airway nerves correlates with the degree of neuronal dysfunction and airway hyperresponsiveness, whereas bronchoalveolar lavage eosinophils do not (Fryer et al. 2006; Nie et al. 2007). Indeed, human and animal studies repeatedly show a persistence of eosinophils in airway tissue despite dramatic reductions in peripheral and lavage eosinophils (Flood‐Page et al. 2003), indicating that the effects of eosinophils must be interpreted in the context of their location within pulmonary compartments. Here, we tested ozone's effects on eosinophils in airway subepithelium and adventitia, and around airway nerves in guinea pigs 1 and 3 days after a single ozone exposure. Differences in eosinophils were characterized in small and large airways, and the ability of antigen sensitization and TNF*α* blockade to alter ozone‐induced eosinophil recruitment to lungs was tested. The relationships between airway, nerve‐associated and bronchoalveolar lavage eosinophils, and ozone‐induced airway hyperresponsiveness were also evaluated.

## Methods

### Animals

Pathogen‐free female Dunkin‐Hartley guinea pigs (Charles River, Kingston, NY) were shipped in filtered crates and housed in high‐efficiency particulate filtered air rooms. Guinea pigs were used due to similarities between human and guinea pig neuro‐anatomy and airway function (Canning and Fischer 1997; Roffel et al. 1997; Kesler and Canning 1999; Tanaka et al. 2005; Kocmalova et al. 2017). Protocols followed NIH guidelines and were approved by the Oregon Heath & Science University Animal Care and Use Committee.

### Ozone exposure

Guinea pigs were exposed to ozone (2.0 ppm) or filtered air for 4 hours in individual wire cages with access to food and water, as previously described (Wicher et al. 2017). Lungs were harvested 1 or 3 days later. Some animals (150–200 g) were sensitized to ovalbumin (4.2 mg i.p. on days 1, 3, and 5) 21 days before exposure to ozone or air. Sensitization was confirmed by the presence of ova‐specific IgE antibodies in bronchoalveolar lavage and lung homogenates using a guinea pig‐specific ova‐IgE ELISA kit (Cusabio, College Park, MD). Some animals were pretreated with a TNF*α* antagonist, etanercept (3mg/kg i.p, Amgen, Thousand Oaks, CA) 3 h before ozone exposure, as previously described (Nie et al. 2009; Proskocil et al. 2013; Wicher et al. 2017).

### Immunohistochemistry

Isolated lungs were fixed in zinc‐buffered formalin (Anatech Ltd., Battle Creek, MI) overnight at 4°C, then stored in 70% ethanol. Transverse sections from left upper and lower lobes were paraffin‐embedded and cut into 10 *μ*m sections. Slides were dewaxed in xylene overnight and rehydrated in 100%, 70%, and 50% ethanol. Antigen retrieval was performed using Antigen Unmasking Solution (Vector, H‐3300, Burlingame, CA). Endogenous peroxidase activity was quenched with 3% H_2_O_2_ in cold methanol (−20°C) and slides were permeabilized in Trypsin (Invitrogen, Carlsbad, CA) for 10 mins at 37°C. DNA was denatured using 2N HCl (30 min) and slides were blocked in 10% normal goat serum (Vector S‐1000, Burlingame, CA). Airway nerves were labeled with pan‐neuronal marker mouse anti‐PGP9 primary antibody (1:250 dilution 4°C overnight; ABD Serotech, Oxford, UK). Goat anti‐mouse biotinylated secondary antibody (Invitrogen) was then applied (1:400 dilution at room temp for 2 hours; Vector, VA‐9200, Burlingame, CA) followed by incubation in Vectastain Elite ABC (Vector, PK‐6100, Burlingame, CA) for 30 mins at room temperature. Slides were developed with Vector SG (Vector). Eosinophils were stained with 1% Chromotrope 2R (Sigma, St. Louis, MO) for 1 min at room temperature. Slides were allowed to dry and mounted in Cytoseal 60 mounting medium (Richard‐Allan Scientific, San Diego, CA).

### Image acquisition

Airway images were obtained with an Apotome.2 wide‐field microscope (Zeiss, Oberkochen, Germany). Using ZEN 2.3 imaging software (Blue Edition, Zeiss), airway coordinates were recorded and a grid was constructed around the outer perimeter of each airway under low power (5x). Individual airways were then imaged at high power (40x) and grid‐based images were stitched together to render a single high‐resolution image for each airway.

### Quantification of airway eosinophils

Airways were analyzed from the respiratory epithelial basement membrane to the tunica adventitia, hereto referred to as “subepithelium” or “airways,” using ImageJ 1.50i (NIH, Bethesda, MD). Only airways with intact subepithelium were included. Small and large airways were analyzed separately based on the presence or absence of cartilage. Cartilage was excluded from calculations of area. Eosinophils in the airway subepithelium were manually counted by a blinded observer, as previously described (Yost et al. 2005; Proskocil et al. 2008). Eosinophils within 8 *μ*m of nerves (the average diameter of an eosinophil) were considered nerve‐associated. Eosinophil counts were normalized to the square millimeters of airway wall.

### Measurement of bronchoalveolar lavage eosinophils

Lungs were lavaged via a tracheal cannula (5 × 10 mL sterile PBS). Total inflammatory cells were counted using a hemocytometer. Eosinophils were quantified from lavage cells cytospun onto slides and stained with hematoxylin and eosin (Hemacolor EMD, Philadelphia, PA).

### Measurement of airway hyperresponsiveness

Guinea pigs were anesthetized with urethane (1.9 g/kg i.p.), paralyzed with succinylcholine (10 *μ*g/kg i.v.), tracheotomized and mechanically ventilated with positive pressure and constant volume (1 mL tidal volume/100 g body wt; 100 breaths/min). Vagus nerves were ligated and distal ends were attached to platinum electrodes. Bronchoconstriction was induced by electrically stimulating both vagus nerves simultaneously (10 V, 0.2 msec pulse width, 25 Hz, 5 sec duration) at 1 min intervals. Bronchoconstriction was calculated as the increase in pulmonary inflation pressure above baseline inflation pressure as previously described (Wicher et al. 2017).

### Statistics

Data are expressed as mean ± SEM. Group means were compared using a one‐way analysis of variance with Bonferroni's post hoc test (Graphpad, La Jolla, CA). Correlations between airway and lavage eosinophils, and between eosinophils and bronchoconstriction, were assessed with using linear regression. A *P* < 0.05 was considered significant. Animals with eosinophil counts greater than 2 standard deviations above the mean were considered outliers and excluded from the final analysis. Three data points met this definition, a single animal from the following groups: nonsensitized 1 day postexposure to air, sensitized 3 days postexposure to air, and sensitized 3 days postexposure to ozone.

## Results

### Subepithelial and nerve‐associated eosinophils are more common in small airways

**Figure 1:**
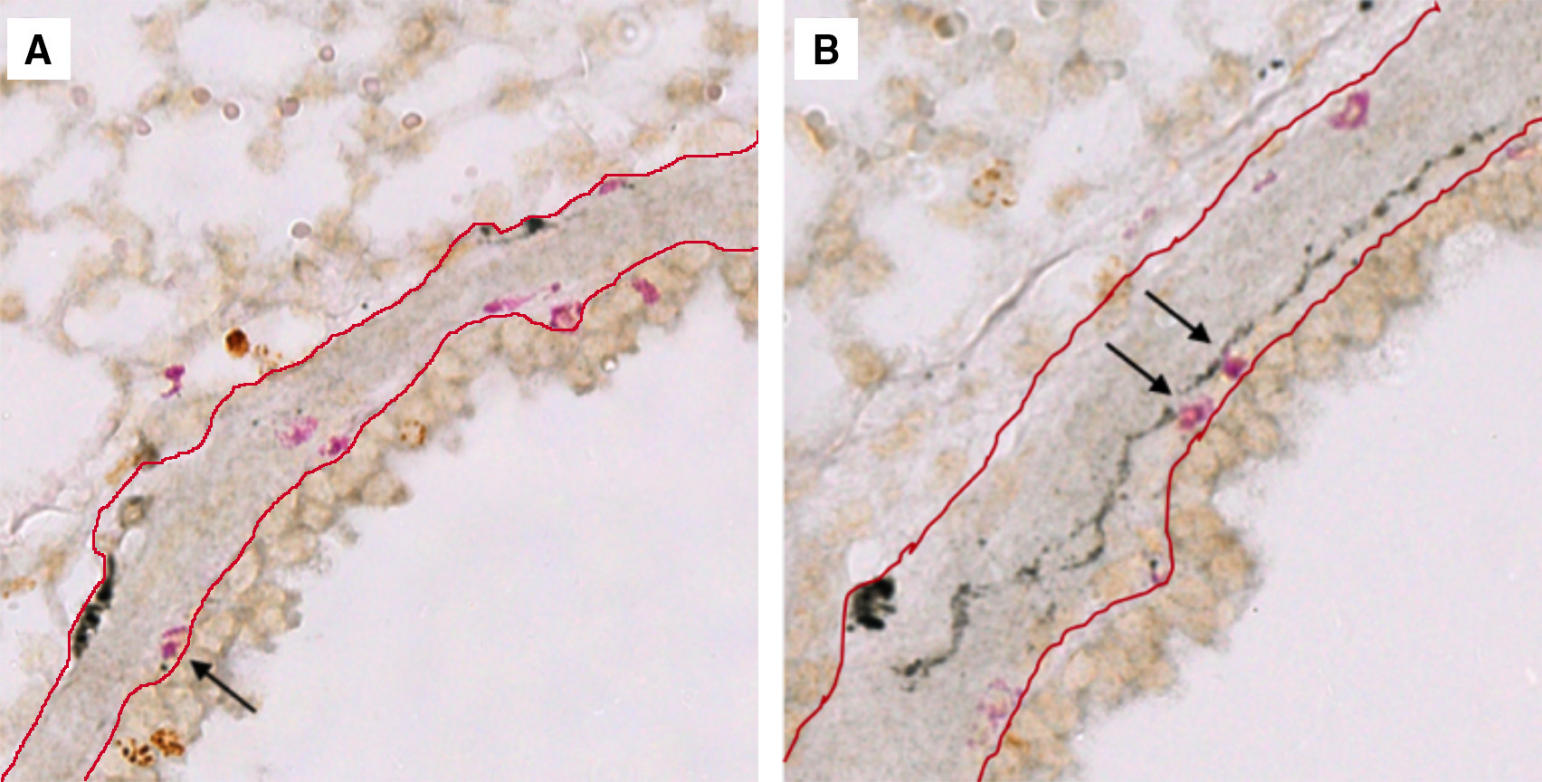
Eosinophils are present in airway subepithelium and along airway nerves. Guinea pig airway nerves (black) and eosinophils (pink) were stained with PGP9.5 and Chromotrope 2R, respectively. Total eosinophils were counted in small, noncartilaginous (A) and large, cartilaginous (B) airways between the epithelial basement membrane and the tunica adventitia (red lines). Eosinophils within 8 *μ*m of nerves were classified as nerve‐associated (arrows).

**Figure 2:**
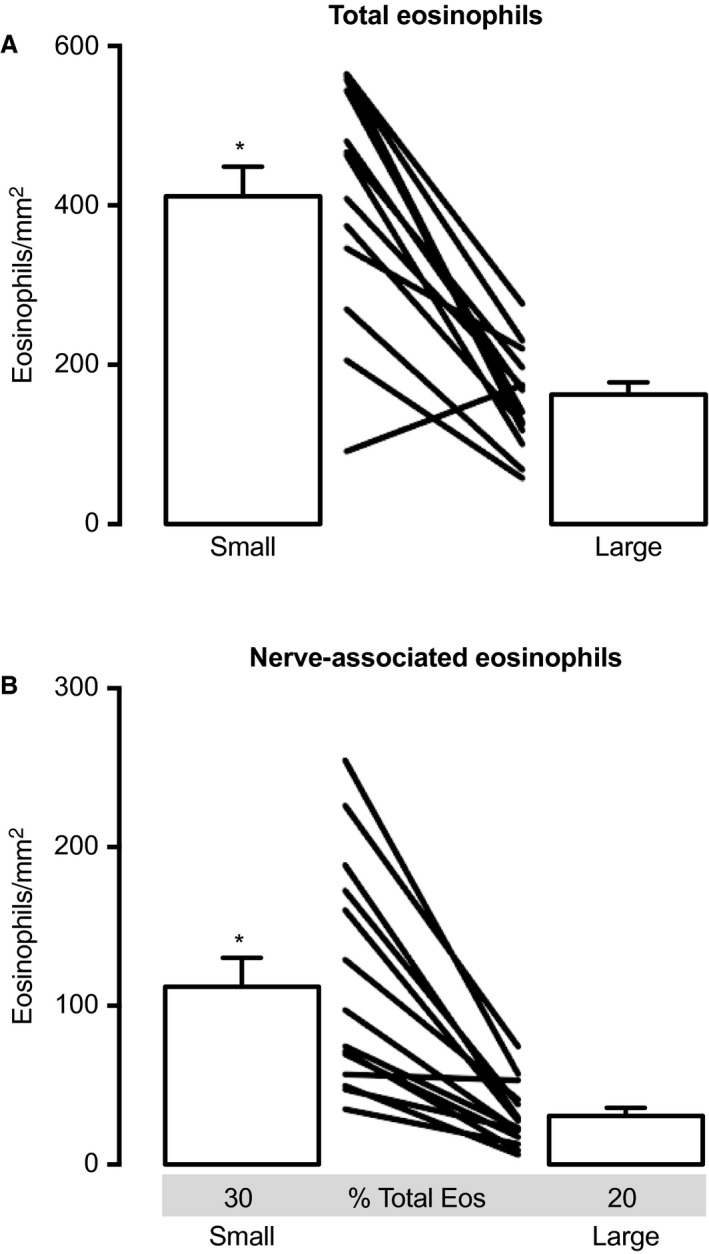
Eosinophils are more numerous in small noncartilaginous airways compared to large cartilaginous airways. (A) Small noncartilaginous airways contained significantly more eosinophils than large cartilaginous airways. This difference was observed in all treatment groups and at all time points after exposure. (B) Small airways also contained more nerve‐associated eosinophils than large airways. A greater proportion of total airway eosinophils were associated with nerves in small airways (30%) compared to large (20%). *N* = 14. Data represent mean ± SEM. *P *< 0.05. Connecting lines distinguish individual animals. All treatment groups are represented in this analysis.

Small, noncartilaginous airways contained significantly more eosinophils than large, cartilaginous airways (411 ± 37 eosinophils/mm^2^ compared to 162 ± 16 eosinophils/mm^2^) (Figs. 1 and 2A). Similarly, more nerve‐associated eosinophils were found in small airways than large (112 ± 18 eosinophils/mm^2^ vs. 31 ± 5) (Fig. 2B). These differences were present 1 and 3 days after air or ozone exposure, regardless of sensitization status or etanercept pretreatment. In small airways approximately 1/3 of all eosinophils were present along nerves, whereas only 1/5 of eosinophils are nerve‐associated in large airways.

### Ozone decreases total airway eosinophils, but not nerve‐associated eosinophils, 3 days later

**Figure 3:**
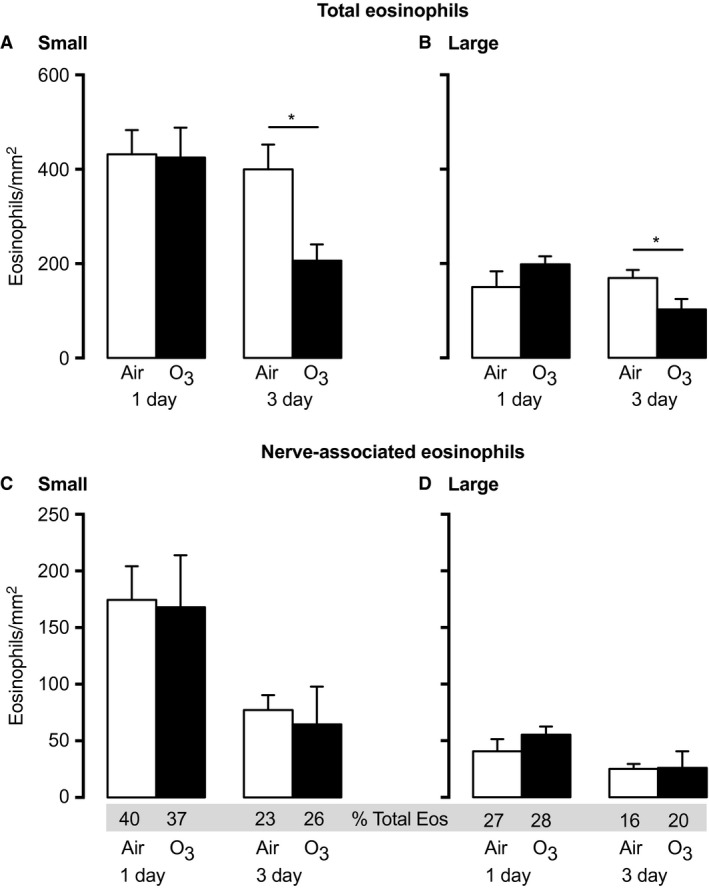
Ozone decreases total airway eosinophils 3 days after exposure in nonsensitized animals. 1 day after ozone, total eosinophils in small (A) or large (B) airways were similar to air‐exposed controls. In contrast, 3 days after ozone, total eosinophils were decreased in both small (A) and large (B) airways. Eosinophils associated with nerves were not different 1 or 3 days after ozone (C and D). *n* = 6–9. Data represent mean ± SEM. *P < 0.05.

One day after ozone, the number of eosinophils in small and large airways were similar to air‐exposed controls. In contrast, 3 days after ozone, total eosinophils were significantly decreased in both small and large airways compared to air‐exposed controls (Fig. 3A–B). The distribution of eosinophils around nerves was unaffected by ozone at either time point in both small and large airways (Fig. 3C–D).

### Ovalbumin sensitization increases nerve‐associated eosinophils in small and large airways

**Figure 4:**
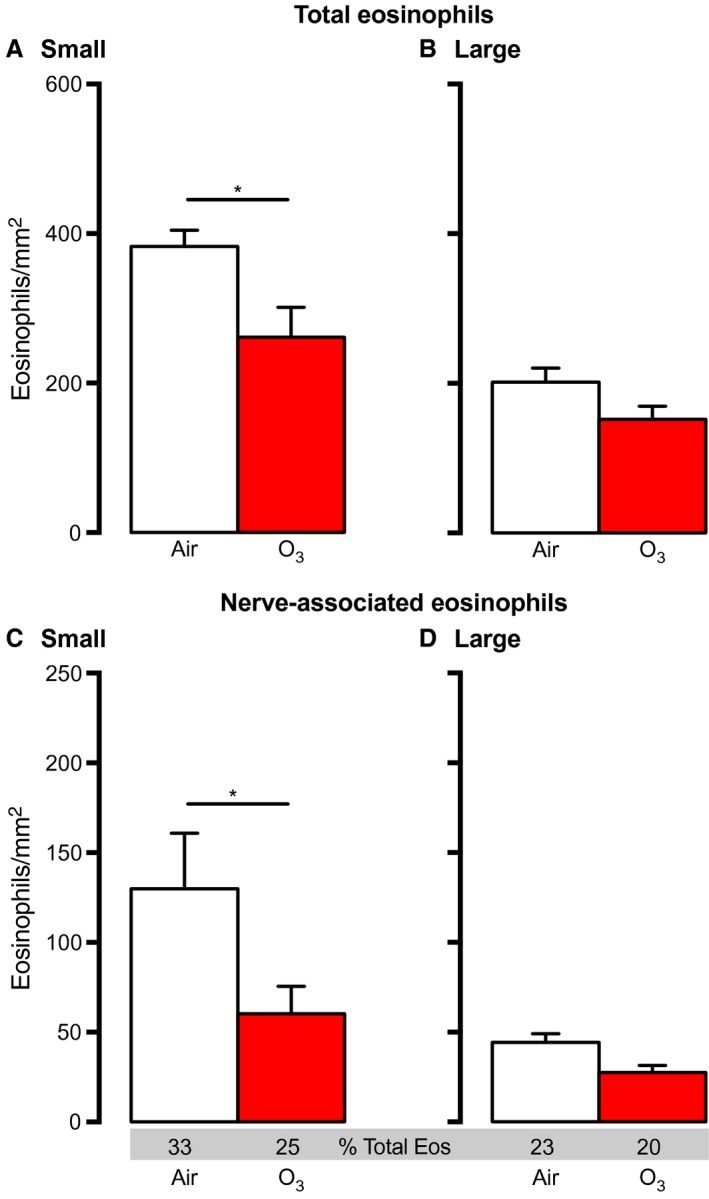
Sensitization alters eosinophil location in lungs 3 days after ozone. Guinea pigs were sensitized to ovalbumin 21 days prior to ozone exposure. Total eosinophils and nerve‐associated eosinophils (absolute number and percent relative to airway eosinophils) were decreased after ozone in small (A and C), but not large (B and D) airways in sensitized animals. *n* = 5–7. Data represent mean ± SEM. *P < 0.05.

Ovalbumin sensitization alone did not alter the total eosinophils in small or large airways (Fig. 4A–B [white bars] vs. Fig. 3A–B 3‐day nonsensitized air [white bars]). However, sensitization alone doubled nerve‐associated eosinophils in both small and large airways (Fig. 4C–D [white bars] versus Fig. 3C–D 3‐day nonsensitized air [white bars]).

### Ozone reduces nerve‐associated eosinophils in sensitized animals

Similar to nonsensitized animals, ozone significantly decreased total eosinophils in small airways of sensitized animals 3 days later (Fig. 4A). A smaller decrease in eosinophils was seen in large airways 3 days after ozone in sensitized animals that did not meet statistical significance (Fig. 4B). Ozone also significantly decreased nerve‐associated eosinophils in small (Fig. 4C) and large (Fig. 4D) airways of sensitized guinea pigs, although again, changes in large airways did not meet statistical significance.

### Etanercept increases airway eosinophils and alters their response to ozone

**Figure 5:**
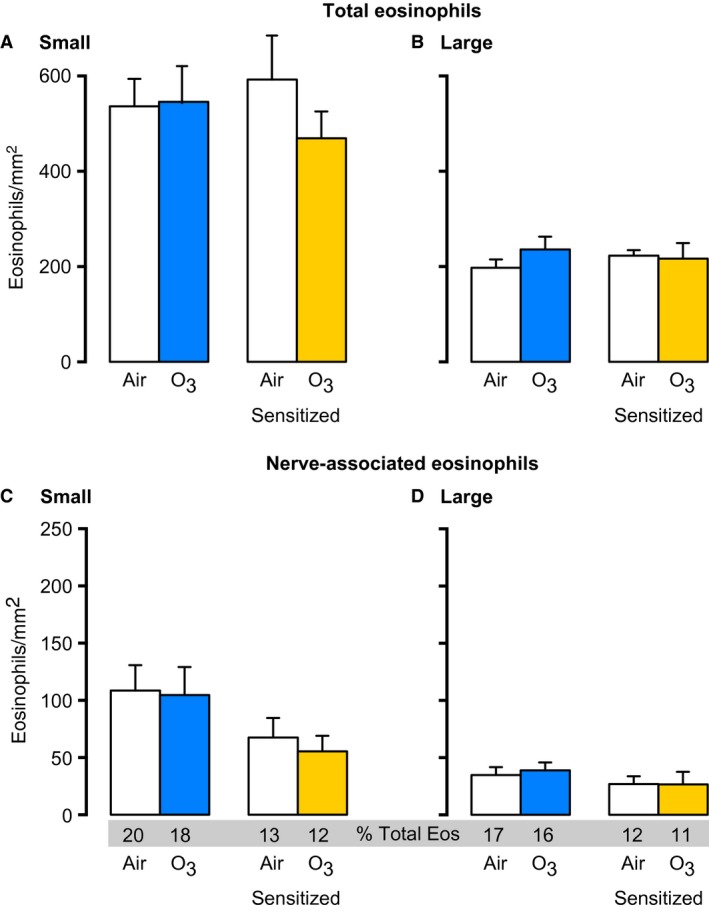
Etanercept prevents airway and nerve‐associated eosinophils from decreasing after ozone. Guinea pigs were pretreated with the TNF *α* antagonist etanercept prior to ozone exposure. Airway eosinophils were measured 3 days later. Etanercept prevented eosinophils in small (A) and large (B) airways from decreasing after ozone in nonsensitized (paired white and blue bars) and ovalbumin‐sensitized animals (paired white and yellow bars). Nerve‐associated eosinophils were similar between air‐ and ozone‐exposed animals in nonsensitized (blue) and sensitized (yellow) groups. *n* = 6–12. Data represent mean ± SEM *P < 0.05

Etanercept alone increased eosinophils in small (Fig. 5A), but not large (Fig. 5B) airways in both nonsensitized and sensitized, air‐exposed control animals. Three days after ozone exposure, etanercept completely blocked the ozone‐induced decrease in small and large airway eosinophils of nonsensitized (paired white and blue bars) and ovalbumin‐sensitized animals (paired white and yellow bars) (Fig. 5A–B). Although nerve‐associated eosinophils were similar between air‐ and ozone‐exposed animals in both nonsensitized and sensitized groups (Fig. 5C–D), nerve‐associated eosinophils as a percent of total airway eosinophils were significantly reduced compared to animals that were not treated with etanercept (Fig. 5C–D vs. Fig. 3C–D nonsensitized 3 day and Fig. 4C–D sensitized).

### Airway eosinophils correlate with airway hyperresponsiveness after ozone

**Figure 6:**
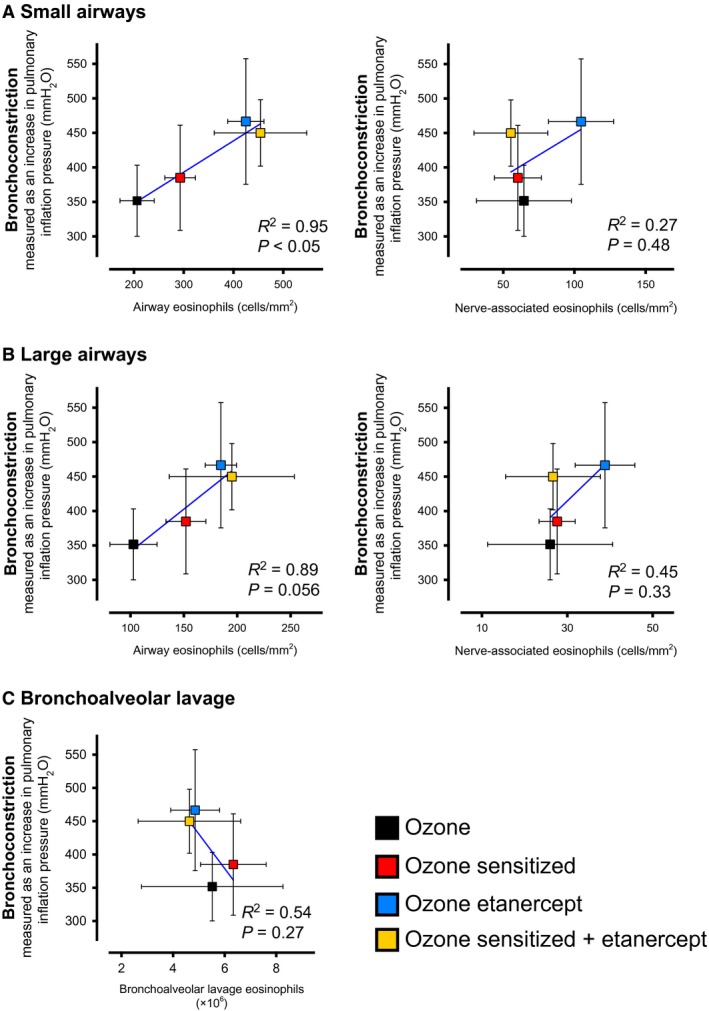
Airway eosinophils, but not nerve‐associated or bronchoalveolar lavage eosinophils, correspond with airway hyperresponsiveness 3 days after ozone. Airway responsiveness to electrical vagal stimulation (25 Hz, 10 V, 0.2 msec) was measured 3 days after ozone in mechanically ventilated guinea pigs. Ozone‐induced (black squares) airway hyperresponsiveness (bronchoconstriction; *y*‐axis) was potentiated by sensitization (red), etanercept (blue) and the combination of both (yellow). The number of small (A) and large (B) airway eosinophils (*x*‐axis) correlated with the severity of airway hyperresponsiveness 3 days after ozone. In contrast, nerve‐associated eosinophils and bronchoalveolar lavage eosinophils (C) did not. Similarly, no correlation between airway responsiveness and eosinophils was observed in any air‐exposed group (data not shown). *n* = 5–10. Data represent means ± SEM.

Bronchoconstriction was measured in response to electrical stimulation of both vagus nerves in anesthetized, paralyzed and mechanically ventilated guinea pigs 3 days after ozone. Ovalbumin sensitization, etanercept treatment and the combination of both increased small and large airway eosinophils, and potentiated ozone‐induced bronchoconstriction 3 days after exposure. At this time point, the number of airway eosinophils for each ozone‐exposed group correlated with the severity of bronchoconstriction (Fig. 6A small airway *R* ^2^ = 0.95, *P* < 0.05, Fig. 6B large airway *R* ^2^ = 0.89, *P* = 0.056). No correlation was found between nerve‐associated eosinophils and bronchoconstriction in either small or large airways (Fig. 6A–B), or between bronchoalveolar lavage eosinophils and bronchoconstriction (Fig. 6C). Similarly, airway, nerve‐associated and bronchoalveolar lavage eosinophils did not correlate with bronchoconstriction in any‐air exposed group (data not shown).

### Changes in airway eosinophils are unrelated to bronchoalveolar lavage eosinophils after ozone exposure

**Figure 7:**
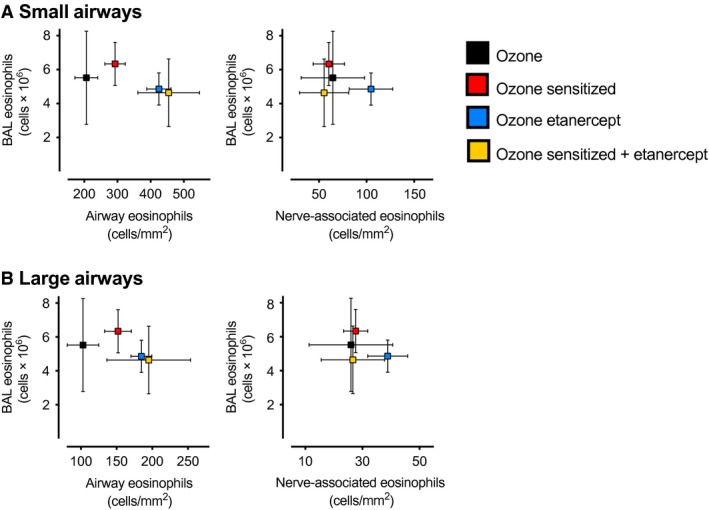
Sensitization and etanercept alter airway eosinophils, but not nerve‐associated or bronchoalveolar lavage eosinophils, 3 days after ozone. Guinea pig bronchoalveolar lavage (BAL) eosinophils and eosinophils within the airway wall were quantified 3 days after ozone. Sensitization (red), etanercept (blue) and the combination of sensitization + etanercept (yellow) blocked a decrease in small (A) and large (B) airway eosinophils after ozone without altering bronchoalveolar lavage eosinophils, indicating eosinophils’ response to ozone differs between distinct airway compartments. Differences between nerve‐associated eosinophils in ozone‐exposed groups were small and were not associated with BAL eosinophils. Neither airway nor nerve‐associated eosinophils correlated with BAL eosinophils in any air‐exposed groups (data not shown). *n* = 5–10. Data represent means ± SEM.

Airway and nerve‐associated eosinophils were compared to bronchoalveolar lavage eosinophils in guinea pigs 3 days after ozone. Etanercept alone, and in combination with sensitization, blocked the ozone‐mediated decrease in airway eosinophils without affecting the number of bronchoalveolar lavage eosinophils (Fig. 7A and B), indicating that ozone's effects on eosinophils are unique to specific airway compartments. Overall, differences between nerve‐associated eosinophils 3 days after ozone were small and were not associated with changes in bronchoalveolar lavage eosinophils. No correlation was found between airway or nerve‐associated eosinophils and bronchoalveolar eosinophils in air‐exposed animals (data not shown).

## Discussion

Distinct eosinophil phenotypes exert dichotomous effects on airway function after ozone exposure. In the acute phase, 1 day after ozone, airway hyperresponsiveness is mediated by mature airway‐resident eosinophils (Wicher et al. 2017). Eosinophils release major basic protein that blocks presynaptic inhibitory M_2_ receptors on parasympathetic nerves, increasing acetylcholine release and bronchoconstriction (Jacoby et al. 1993; Costello et al. 1997, 1999; Yost et al. 1999, 2005). However, over the course of 3 days after ozone exposure, the role of eosinophils changes due to an influx of newly divided, bone marrow‐derived eosinophils into the airways (Wicher et al. 2017). During this second phase, newly‐divided eosinophils attenuate airway hyperresponsiveness. Depleting these “protective” eosinophils significantly worsens bronchoconstriction (Yost et al. 1999, 2005; Wicher et al. 2017). During both phases, eosinophils are found around airway nerves. Nerve function correlates with the number of eosinophils in close proximity to nerves, but not eosinophils in bronchoalveolar lavage (Evans et al. 2001; Fryer et al. 2006; Nie et al. 2007). Thus, ozone's effects on eosinophils in airways and around airway nerves are highly relevant to airway hyperresponsiveness after ozone exposure.

Here, we measured airway and nerve‐associated eosinophils in small noncartilaginous airways and in large cartilaginous airways separately, since nerve density decreases from proximal to distal airways (Scott et al. 2014). Small airways were characterized by more eosinophils overall, and more nerve‐associated eosinophils compared to large airways. This difference was observed in all treatment groups, including in controls, which suggests that eosinophils are recruited to small and large airways via distinct mechanisms. Eosinophils’ effects on small airway nerves may be particularly relevant to asthma given that small airways contribute disproportionately to clinical airflow obstruction (Yanai et al. 1992). That said, the relative contributions of small versus large airway nerves to asthma‐related pathophysiology are not known. The substantial difference between small and large airway eosinophils in our study underscores the importance of including airway size in future analyses.

Depleting eosinophils with an antibody to IL‐5, or blocking eosinophil major basic protein prevents ozone‐induced airway hyperresponsiveness 1 day after exposure by protecting M_2_ receptor function and restoring regulation of parasympathetic nerve acetylcholine release (Yost et al. 1999, 2005). Mature, airway resident eosinophils mediate ozone's acute effects at this time point since eosinophils in bronchoalveolar lavage do not label with BrdU, indicating they have not recently divided (Wicher et al. 2017). Additionally, blocking eosinophil recruitment into lungs does not prevent acute ozone‐induced airway hyperresponsiveness (Yost et al. 2005). Our findings are consistent with these data. Although the number of airway eosinophils or nerve‐associated eosinophils did not change 1 day after ozone, more eosinophils were associated with nerves at this early time point compared to 3 days later (37% vs. 26% in small airways and 28% vs. 20% in large airways), indicating eosinophils remain clustered along nerves where they induce M_2_ dysfunction and airway hyperresponsiveness.

Eosinophils are also found clustered along airway nerves in humans with fatal asthma, and in a variety of experimental animal models of asthma (Costello et al. 1997; Yost et al. 2005). Nerves release tachykinins (Numao and Agrawal 1992; Dunzendorfer and Wiedermann 2000) and eotaxin (Sawatzky et al. 2002; Fryer et al. 2006; Nie et al. 2012) that recruit eosinophils, and express adhesion molecules, such as ICAM‐1 and VCAM‐1 that bind to eosinophil surface receptors (Sawatzky et al. 2002; Yost et al. 2005; Nie et al. 2012). Binding prompts eosinophil release of major basic protein and other cationic granule proteins, neurotrophins, and growth factors that affect nerve function, growth and survival (Lee et al. 2001; Kobayashi et al. 2002). In turn, neuronal M_2_ muscarinic receptors are dysfunctional in airways of asthmatics and in animals after acute exposure to ozone (Schultheis et al. 1994; Yost et al. 1999, 2005), sensitization and challenge with antigen (Costello et al. 1997; Nie et al. 2009), infection with parainfluenza virus (Adamko et al. 1999; Rynko et al. 2014), exposure to increased circulating insulin (Nie et al. 2014) and exposure to organophosphate pesticides (Proskocil et al. 2010).

By 3 days after ozone, newly divided eosinophils arrive in the lungs and reduce airway hyperresponsiveness (Wicher et al. 2017). At this time point, depleting eosinophils with AbIL5, or blocking eosinophil migration into lungs with antibody against very late antigen‐4 (VLA4) substantially worsens airway hyperresponsiveness (Yost et al. 2005). Despite a significant increase in new eosinophils in bronchoalveolar lavage between day 1 and 3, we found the number of nerve‐associated eosinophils does not change. However, our analysis did not assess nerve‐associated eosinophils on day 2, which previous studies have shown is when nerve‐associated eosinophils reach a nadir after ozone (Murlas and Roum 1985; Schultheis and Bassett 1994; Villegas‐Castrejon et al. 1999; Yost et al. 2005). Thus, by day 3, the number of nerve‐associated eosinophils is actually increasing. This increase is likely driven by the arrival of newly divided, protective eosinophils that restore nerve function. Restoring neuronal M_2_ muscarinic receptor function on day 3, though, does not fully reverse airway hyperresponsiveness (Verhein et al. 2011). Unlike at 1 day, both neuronal and nonneuronal mechanisms account for ozone‐induced airway hyperresponsiveness 3 days later (Yost et al. 2005). Similarly, our data show that unlike day 1, the number of airway eosinophils, but not nerve‐associated eosinophils, corresponds with airway hyperresponsiveness on day 3. Ozone's effects are mediated by multiple signals at this time point, including nerve growth factor, substance P and IL‐1*β* (Verhein et al. 2008, 2011). Blocking each independently prevented ozone‐mediated airway hyperresponsiveness 3 days later.

Eosinophils’ ability to attenuate ozone's effects are in line with several recent studies that redefine eosinophils as complex regulators of immune polarization, not simply harmful effector cells in the airways. Indeed, eosinophils are capable of producing lipoxins and resolvins that suppress inflammation, and may interact with a variety of resident airway cells (i.e. macrophages, T cells, etc.) that contribute to lung repair (Lee et al. 2010; Jacobsen et al. 2014). The majority of these studies have focused on allergen challenge models in mice. Far less is known about the mechanisms that govern eosinophil‐mediated resolution of inflammation after ozone. Future studies are needed to determine the phenotype and cytokine profile of protective eosinophils after ozone.

Ozone‐induced inflammatory responses differ between nonatopic and atopic asthmatics, and between nonsensitized and antigen sensitized animals. Specifically, atopic asthmatics and sensitized animals develop a more robust airway eosinophilia detected in bronchoalveolar lavage (Peden et al. 1995; Khatri et al. 2009; Hernandez et al. 2010, 2012; Shim et al. 2015). Sensitization alone, independent of ozone, also increases nerve‐associated eosinophils (33% in sensitized vs. 23% in nonsensitized). However, sensitization does not independently cause airway hyperresponsiveness. Eosinophils recruited to nerves following sensitization still require a stimulus, such as ozone, to induce nerve dysfunction. Unlike in nonsensitized animals, ozone‐induced airway hyperresponsiveness in sensitized animals is entirely eosinophil‐dependent 3 days later (Wicher et al. 2017). Beneficial effects of eosinophils do not occur because sensitization blocks ozone‐induced eosinophil hematopoiesis. As a result, newly divided, protective eosinophils fail to develop and their contribution to repairing airway function after ozone is completely lost (Wicher et al. 2017).

Eosinophil hematopoiesis and recruitment of newly divided, protective eosinophils to airways after ozone is paradoxically regulated by the pro‐inflammatory cytokine TNF*α* (Wicher et al. 2017). Blocking TNF*α* with etanercept prevents expansion of eosinophils in the bone marrow and potentiates ozone‐induced airway hyperresponsiveness. We found etanercept also prevents airway eosinophils from decreasing 3 days after ozone. Since etanercept blocks generation of new eosinophils, our findings suggest persistent eosinophilia in etanercept‐treated animals is due to retention of mature eosinophils in airways that cause ongoing hyperresponsiveness. Our data also suggest TNF*α* has a homeostatic function by regulating eosinophils at baseline. More airway and nerve‐associated eosinophils were present in nonsensitized, air‐exposed control animals treated with etanercept, indicating TNF*α* suppresses the number of baseline eosinophils irrespective of ozone exposure.

TNF*α*'s role is altered by antigen sensitization. Sensitization prevents generation of new protective eosinophils and potentiates ozone‐induced hyperresponsiveness. Blocking TNF*α* in sensitized animals has no additional effect on airway physiology (Wicher et al. 2017). Thus, TNF*α* no longer regulates new eosinophils after ozone in sensitized animals. However, similar to nonsensitized animals, etanercept prevented airway and nerve‐associated eosinophils from decreasing 3 days after ozone, and also increased small airway eosinophils in sensitized animals exposed to air. Therefore, it is likely TNF*α* continues to regulate mature airways eosinophils in sensitized animals, both at baseline and after ozone, similar to nonsensitized animals.

TNF*α* antagonists have produced variable results in humans with asthma (Rouhani et al. 2005; Berry et al. 2006; Brightling et al. 2008; Wenzel et al. 2009; Bice et al. 2014). In those studies, TNF*α* blockade did not alter eosinophilic inflammation in sputum or bronchoalveolar lavage. Our study highlights the limitations of interpreting the effects of TNF*α* antagonists on eosinophils using this strategy. In our study and others (Haldar et al. 2009), bronchoalveolar lavage eosinophils do not correlate with histologic assessments of airway or nerve‐associated eosinophils. Furthermore, our data suggest the benefits of TNF*α* antagonists in asthma may depend on atopic status. Recognizing that TNF*α*'s role may change in nonatopic versus atopic asthmatics could improve analyses of TNF*α* antagonist treatment responses in future clinical studies.

In sum, we show ozone induces distinct changes in airway and nerve‐associated eosinophils 3 days after exposure that are not reflected by changes in bronchoalveolar lavage eosinophils. Specifically, airway eosinophils, but not nerve‐associated eosinophils, decrease by day 3, which corresponds with improvements in airway hyperresponsiveness. TNF*α* mediates ozone's effects on airway eosinophils only in nonsensitized animals. Sensitization alters ozone's mechanisms and potentiates ozone‐induced airway hyperresponsiveness. Blocking TNF*α* in sensitized animals has no effect. Our results suggest future human studies should consider the atopic status of patients, as well as eosinophil phenotype and their location within specific airway compartments, including around airway nerves, when evaluating mechanisms of ozone‐induced airway hyperresponsiveness. A better understanding of these contributing factors is crucial to the successful future of personalized therapy.

## Conflicts of Interest

The authors have no conflicts of interest to report.
